# Supplementary figures and images for: Connect attack in IoT-WSN detect through cyclic analysis based on forward and backward elimination
Source: PeerJ Comput Sci. 2024 Jun 28;10:e2130. doi: 10.7717/peerj-cs.2130 (PMC11232611; doi:10.7717/peerj-cs.2130)

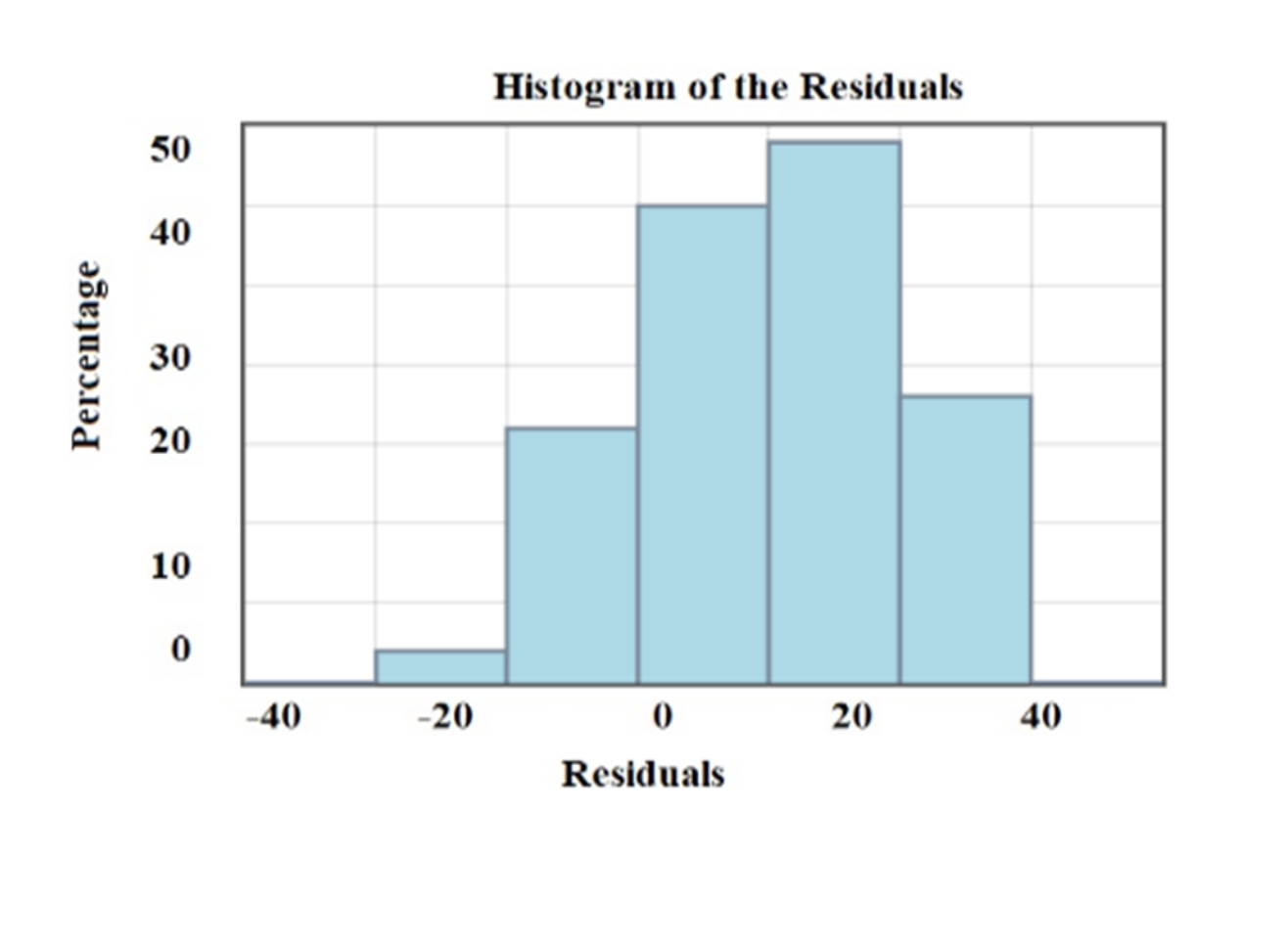

Supplement: Supplemental Information 1 [file peerj-cs-10-2130-s001.zip › Figure S1 Forward Selection CAM StaffSS/Fig S1 Forward Selection Approach/k.png]

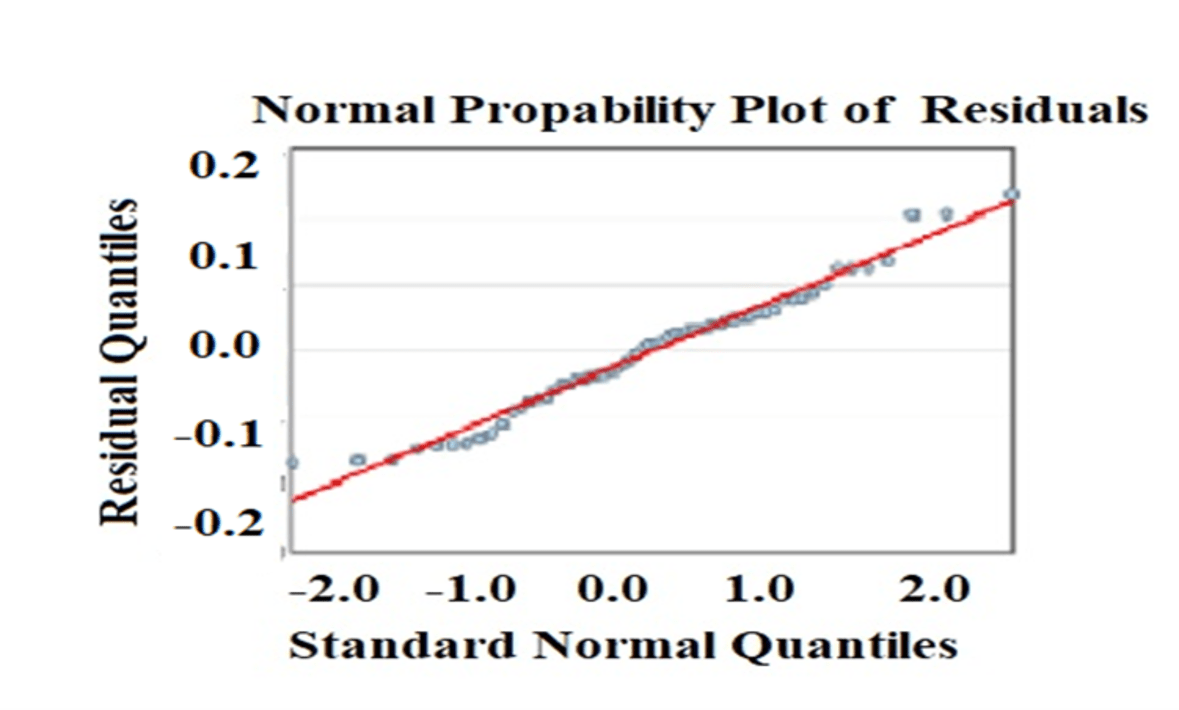

Supplement: Supplemental Information 1 [file peerj-cs-10-2130-s001.zip › Figure S1 Forward Selection CAM StaffSS/Fig S1 Forward Selection Approach/j.png]

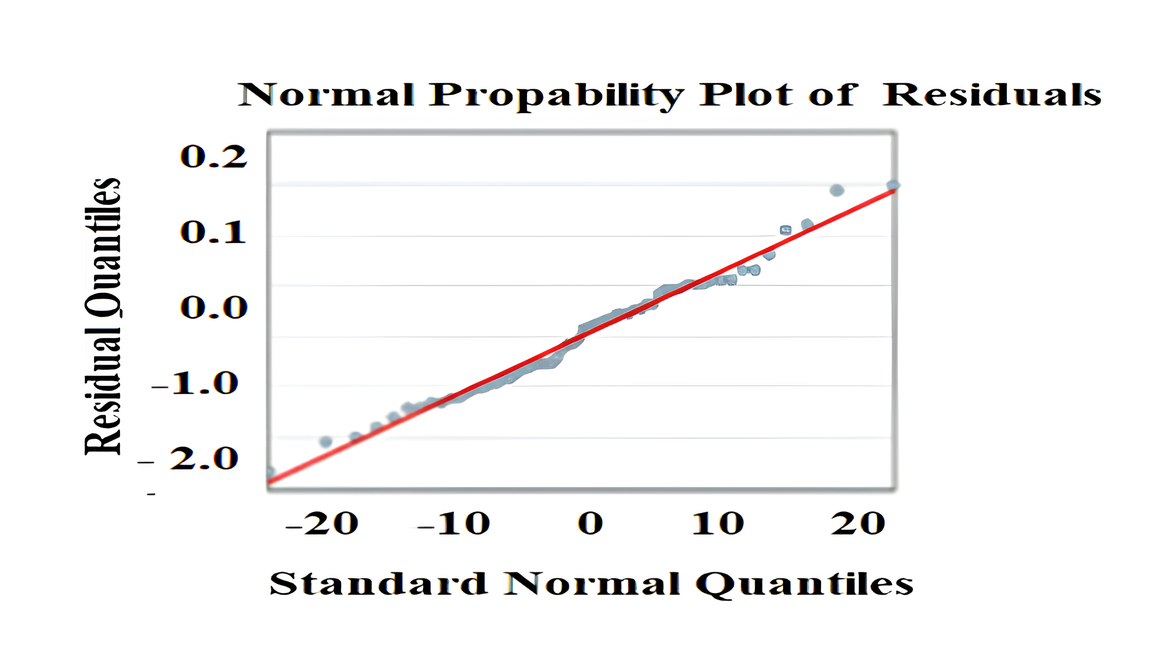

Supplement: Supplemental Information 1 [file peerj-cs-10-2130-s001.zip › Figure S1 Forward Selection CAM StaffSS/Fig S1 Forward Selection Approach/h.png]

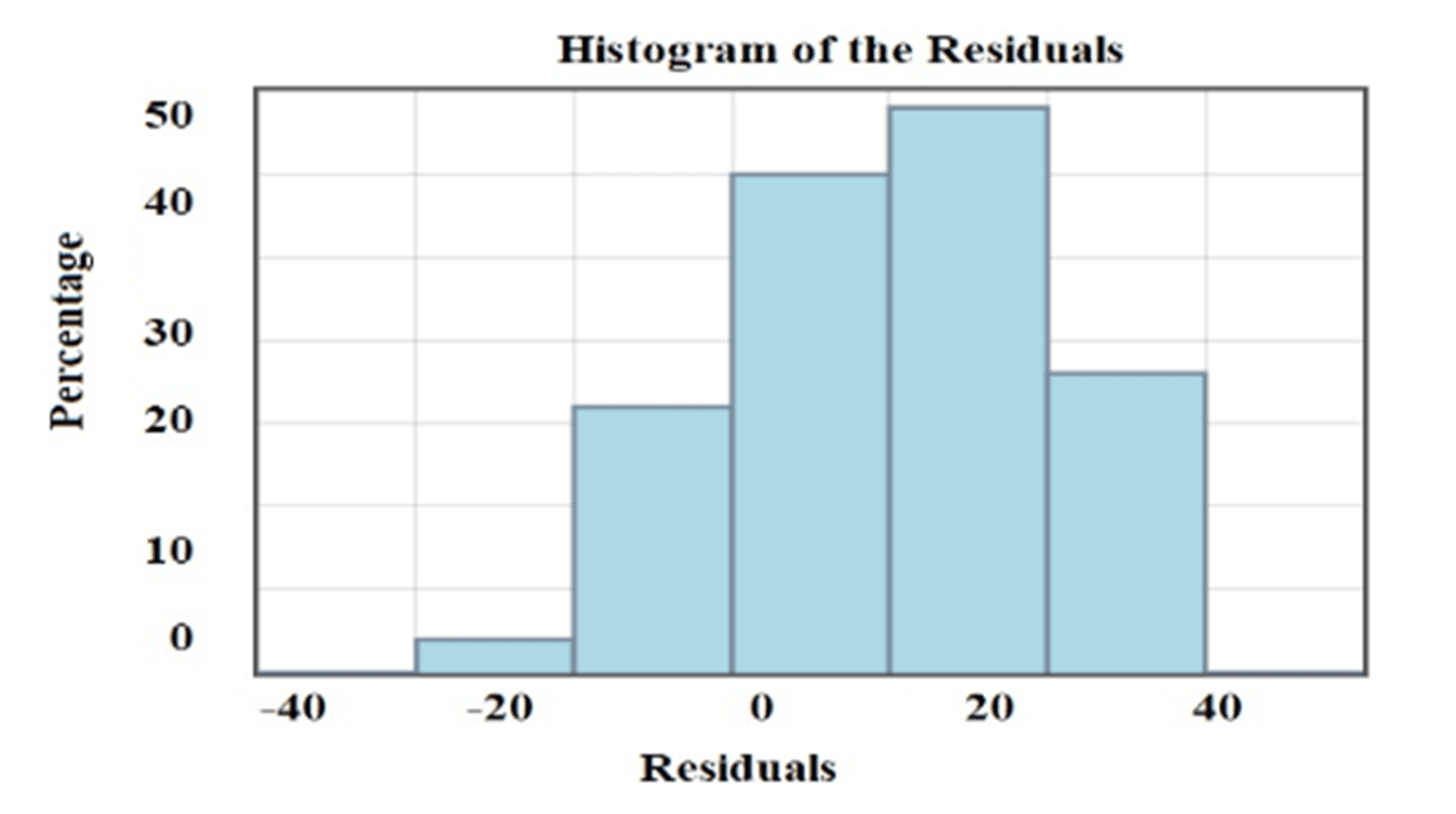

Supplement: Supplemental Information 1 [file peerj-cs-10-2130-s001.zip › Figure S1 Forward Selection CAM StaffSS/Fig S1 Forward Selection Approach/i.png]

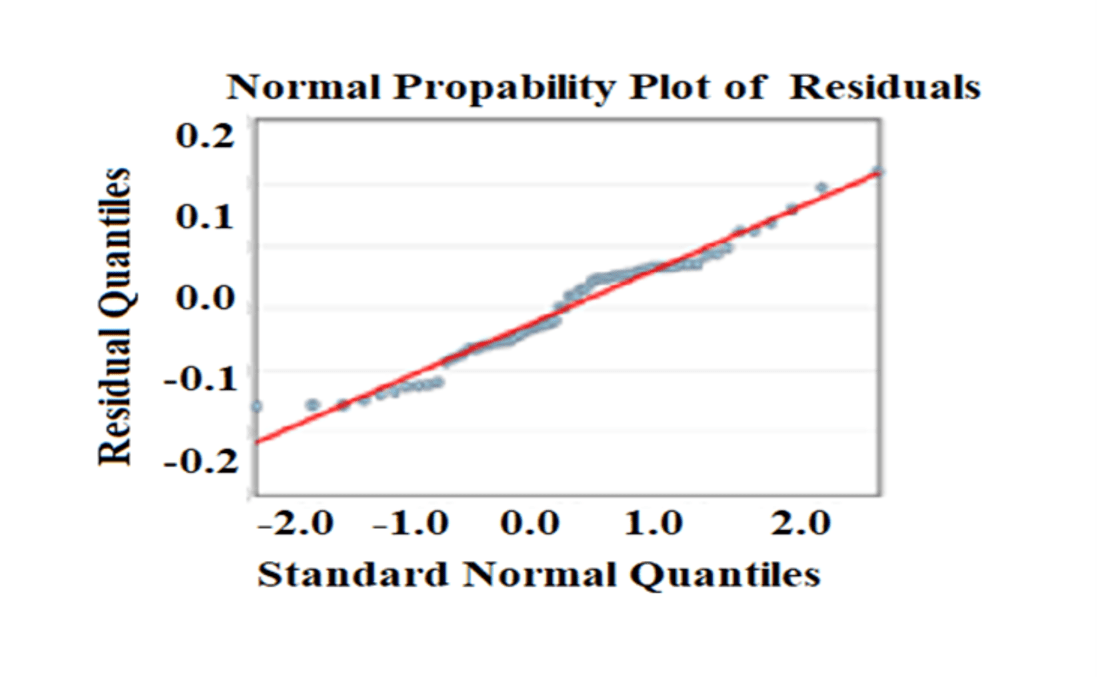

Supplement: Supplemental Information 1 [file peerj-cs-10-2130-s001.zip › Figure S1 Forward Selection CAM StaffSS/Fig S1 Forward Selection Approach/l.png]

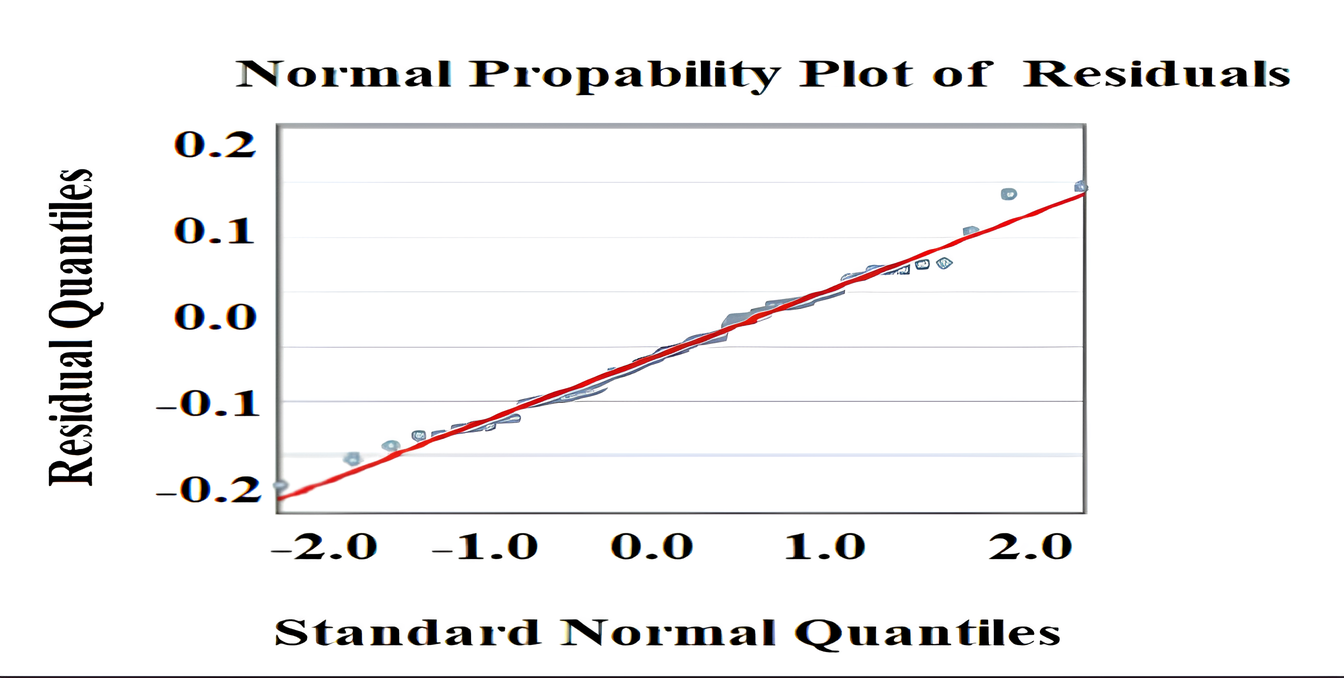

Supplement: Supplemental Information 1 [file peerj-cs-10-2130-s001.zip › Figure S1 Forward Selection CAM StaffSS/Fig S1 Forward Selection Approach/b.png]

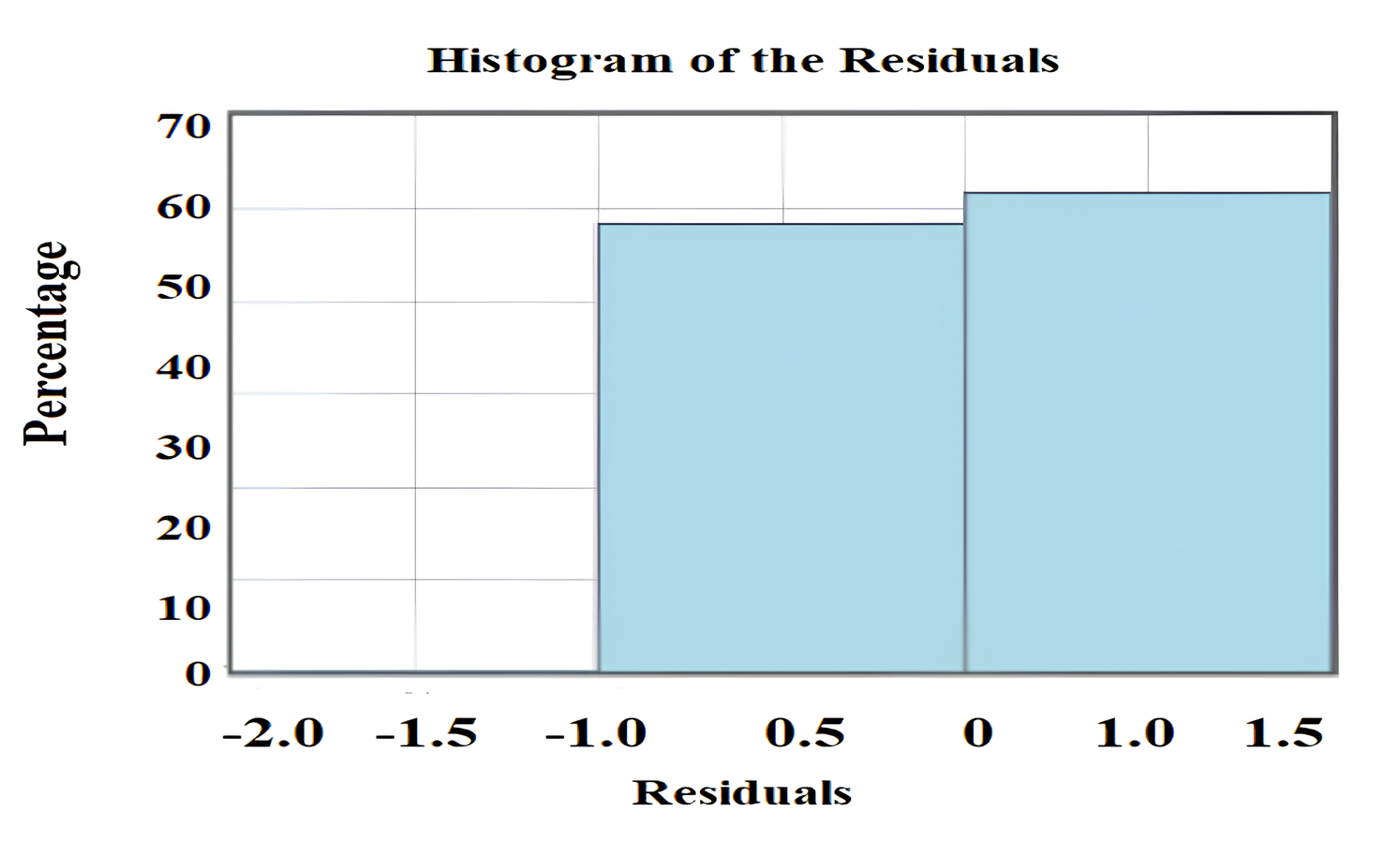

Supplement: Supplemental Information 1 [file peerj-cs-10-2130-s001.zip › Figure S1 Forward Selection CAM StaffSS/Fig S1 Forward Selection Approach/c.png]

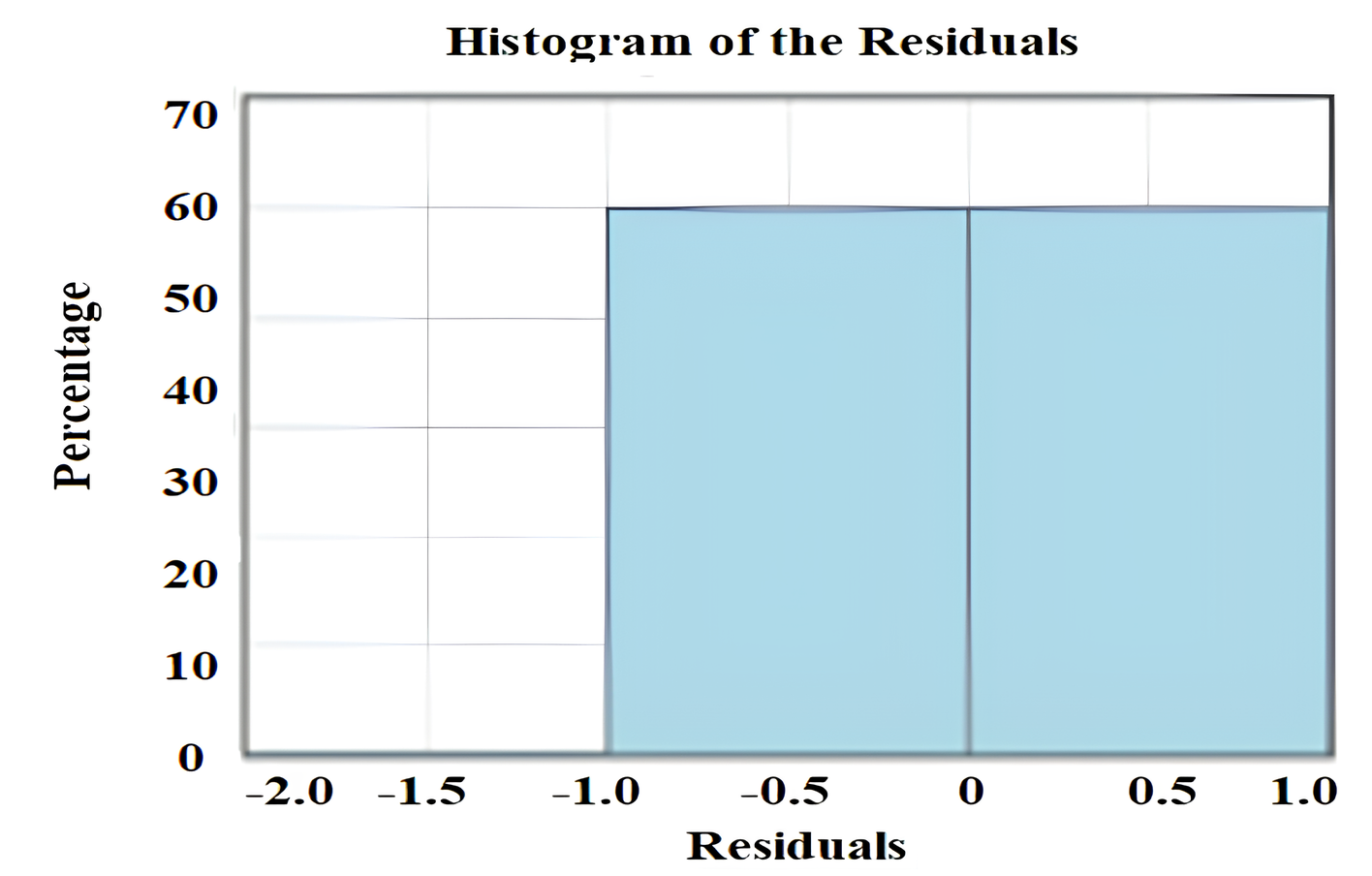

Supplement: Supplemental Information 1 [file peerj-cs-10-2130-s001.zip › Figure S1 Forward Selection CAM StaffSS/Fig S1 Forward Selection Approach/a.png]

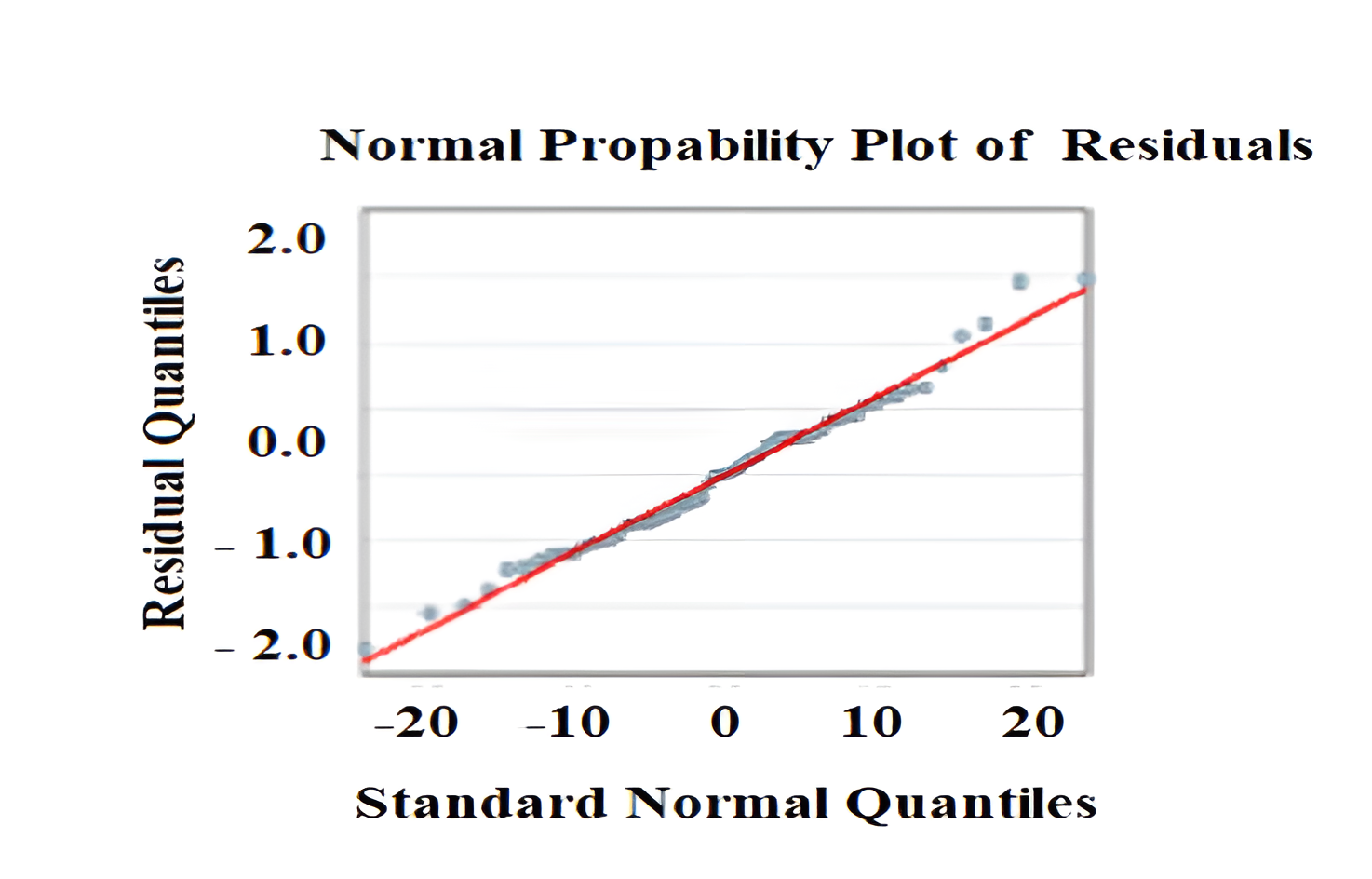

Supplement: Supplemental Information 1 [file peerj-cs-10-2130-s001.zip › Figure S1 Forward Selection CAM StaffSS/Fig S1 Forward Selection Approach/d.png]

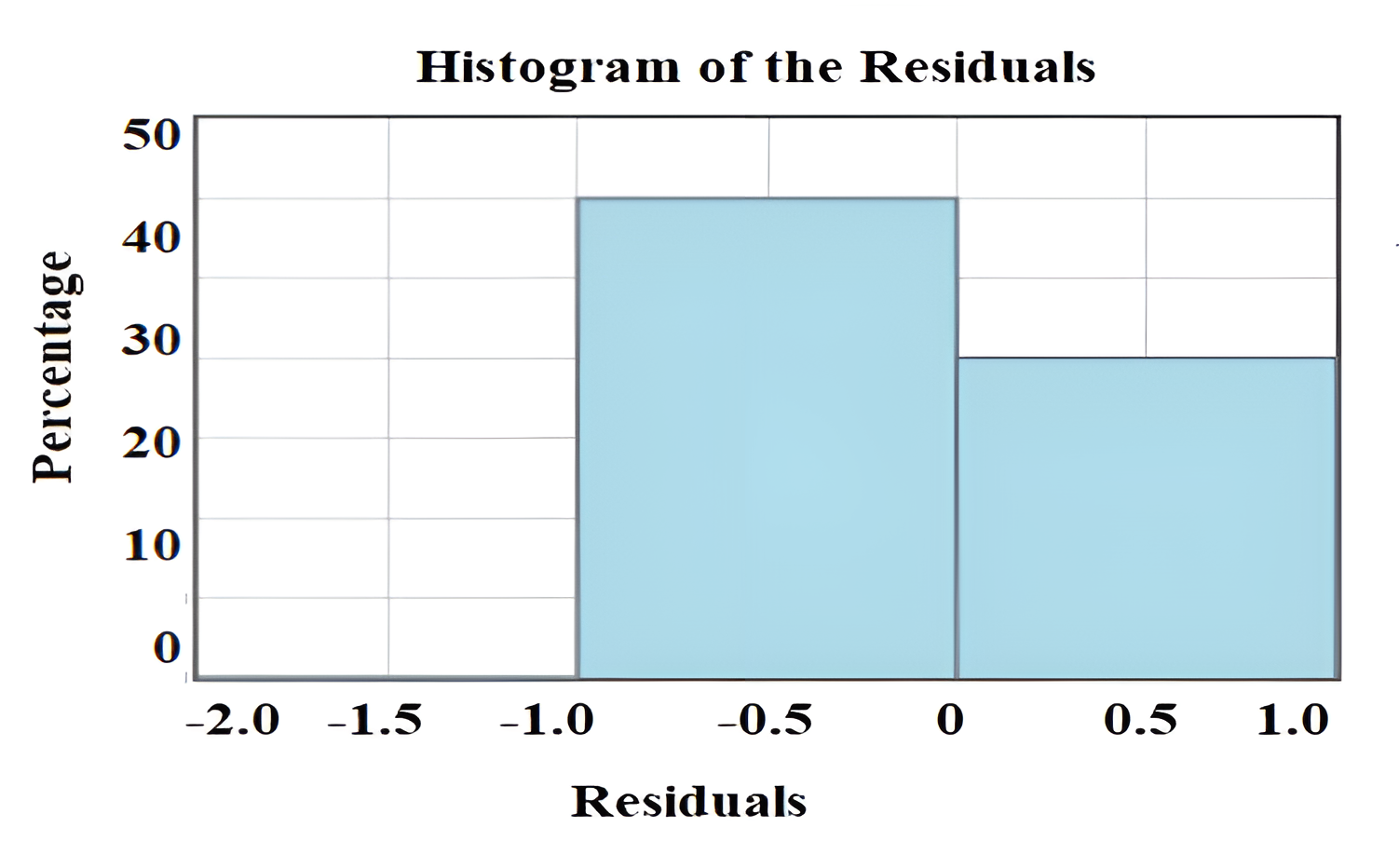

Supplement: Supplemental Information 1 [file peerj-cs-10-2130-s001.zip › Figure S1 Forward Selection CAM StaffSS/Fig S1 Forward Selection Approach/e.png]

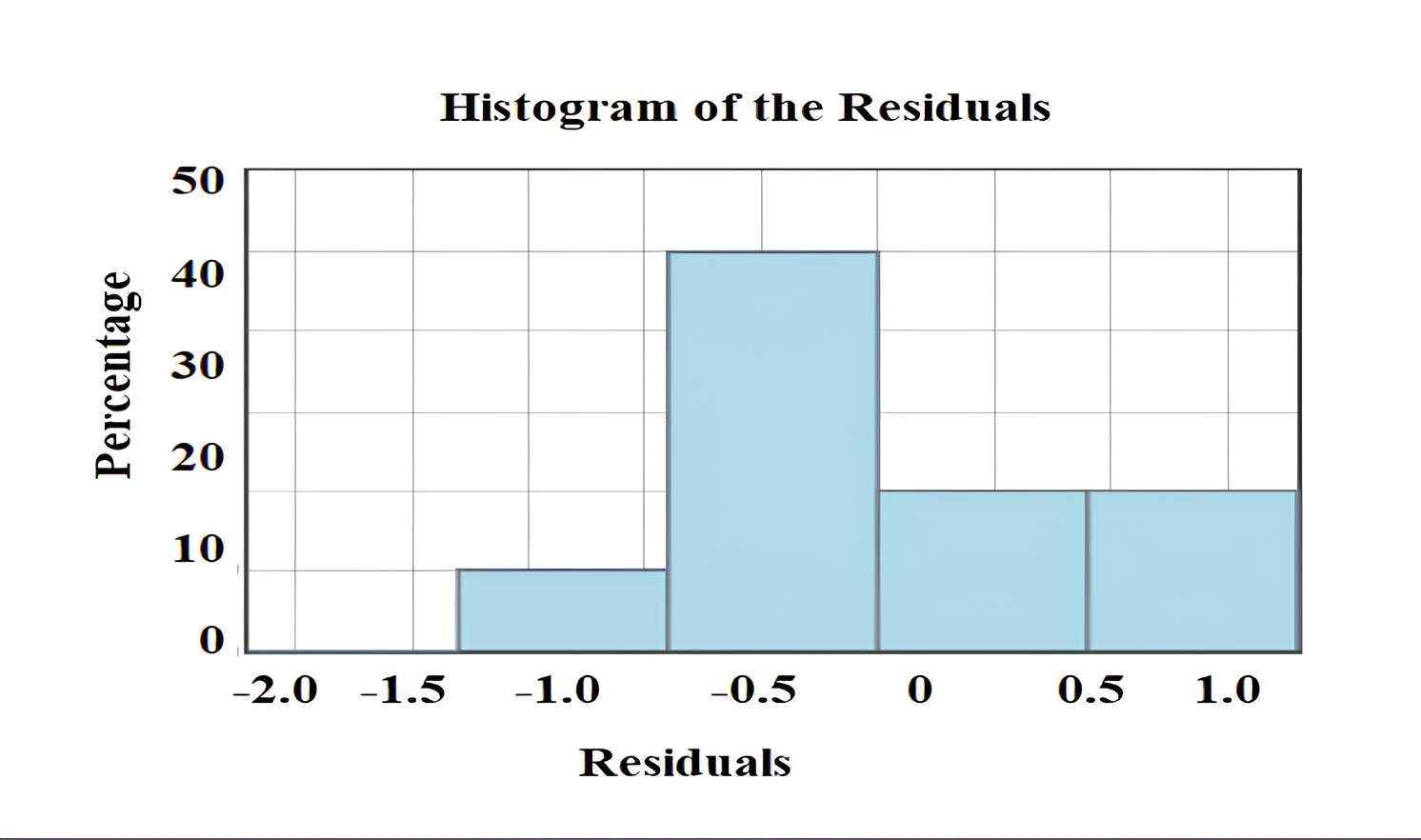

Supplement: Supplemental Information 1 [file peerj-cs-10-2130-s001.zip › Figure S1 Forward Selection CAM StaffSS/Fig S1 Forward Selection Approach/g.png]

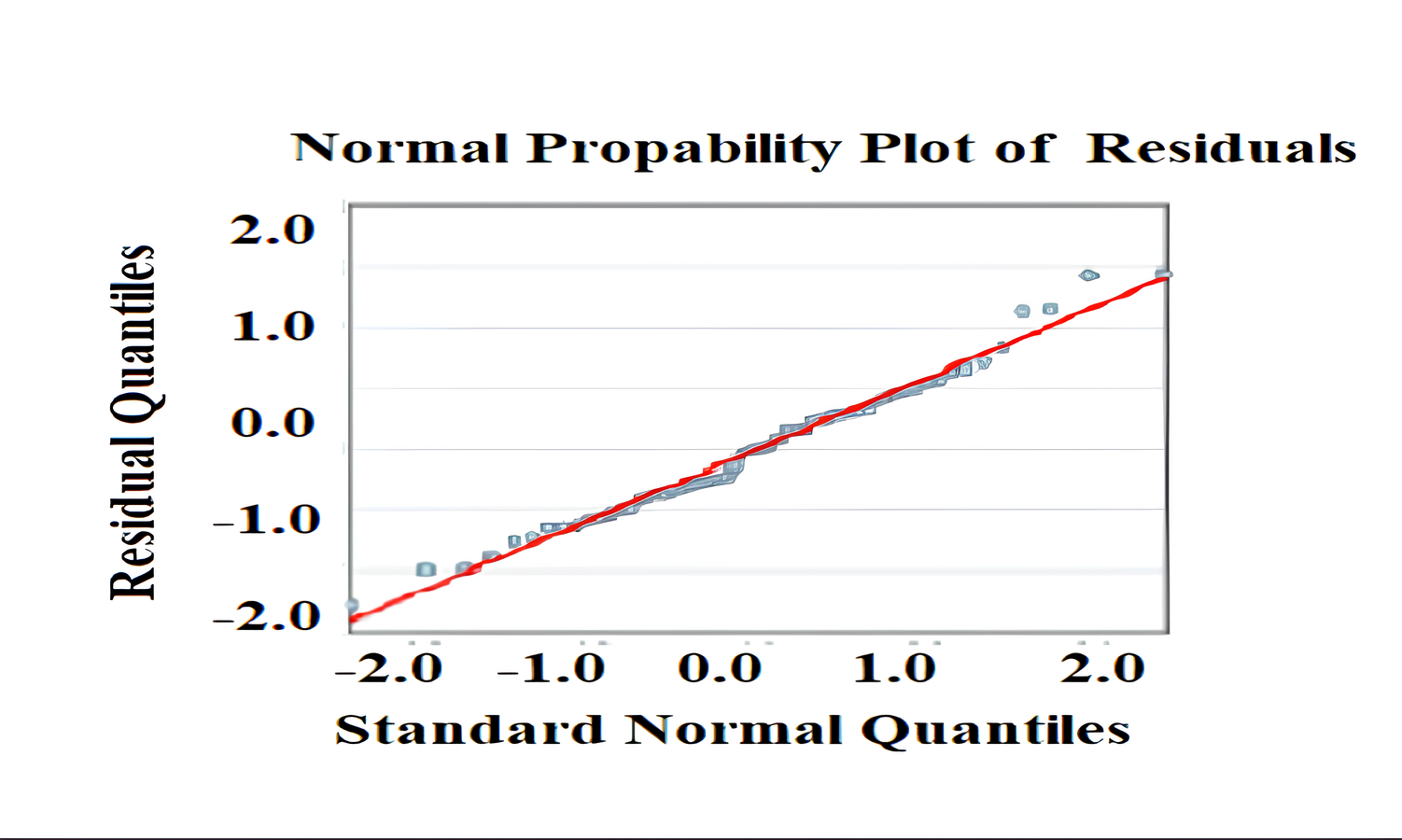

Supplement: Supplemental Information 1 [file peerj-cs-10-2130-s001.zip › Figure S1 Forward Selection CAM StaffSS/Fig S1 Forward Selection Approach/f.png]

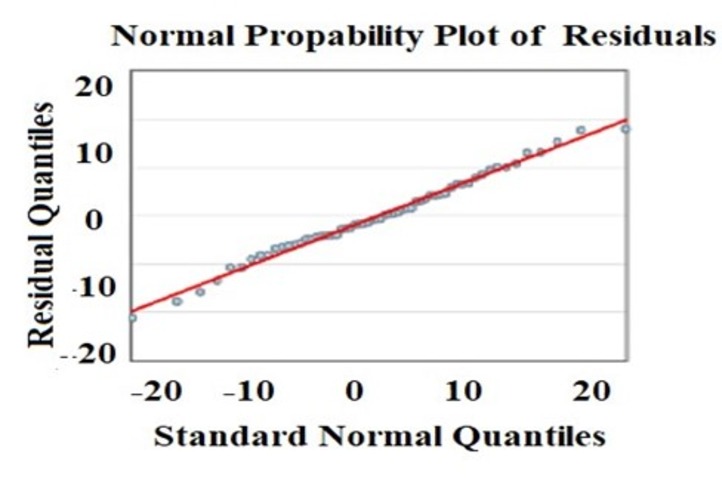

Supplement: Supplemental Information 2 [file peerj-cs-10-2130-s002.zip › Figure S2 Backward Elimination CAM StaffSS/h.jpg]

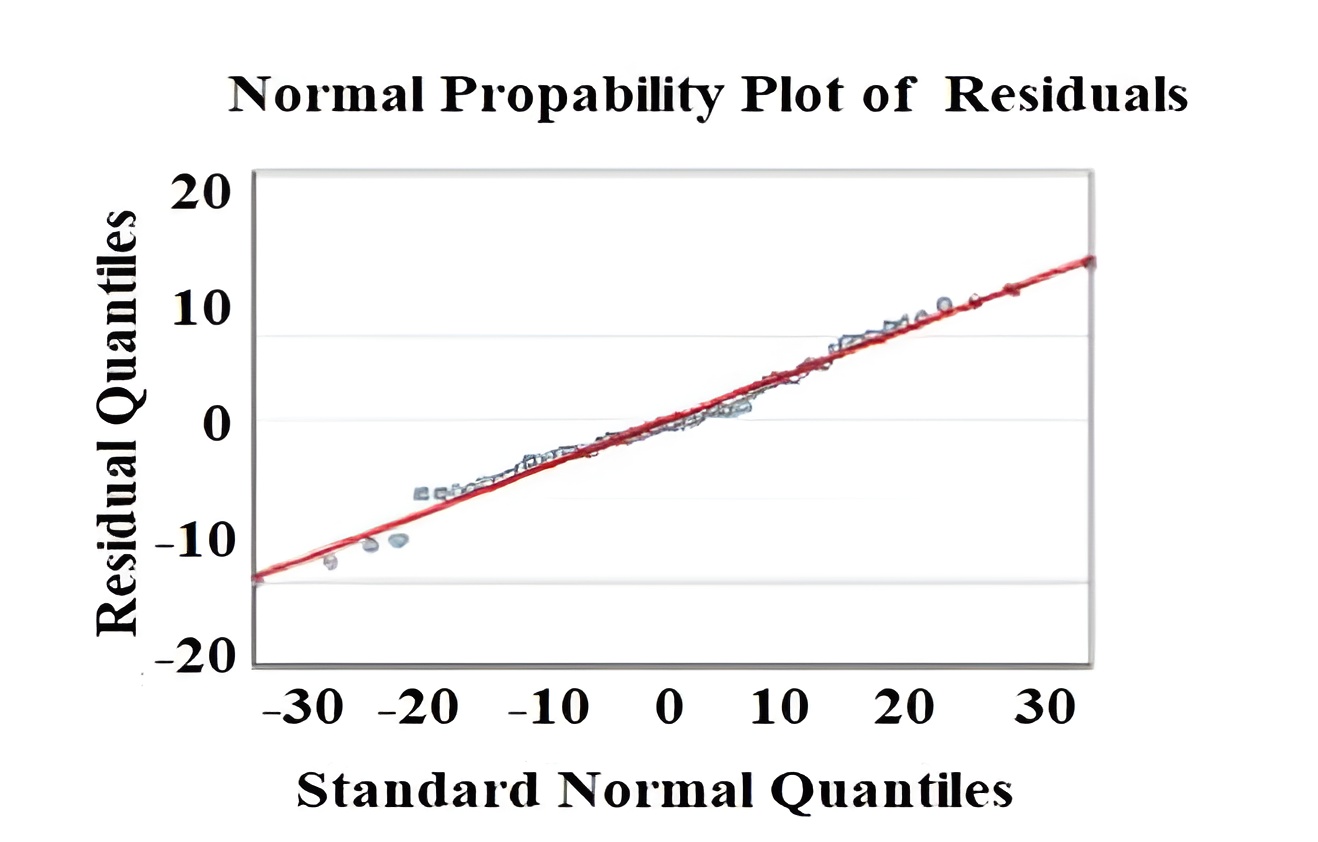

Supplement: Supplemental Information 2 [file peerj-cs-10-2130-s002.zip › Figure S2 Backward Elimination CAM StaffSS/b.jpg]

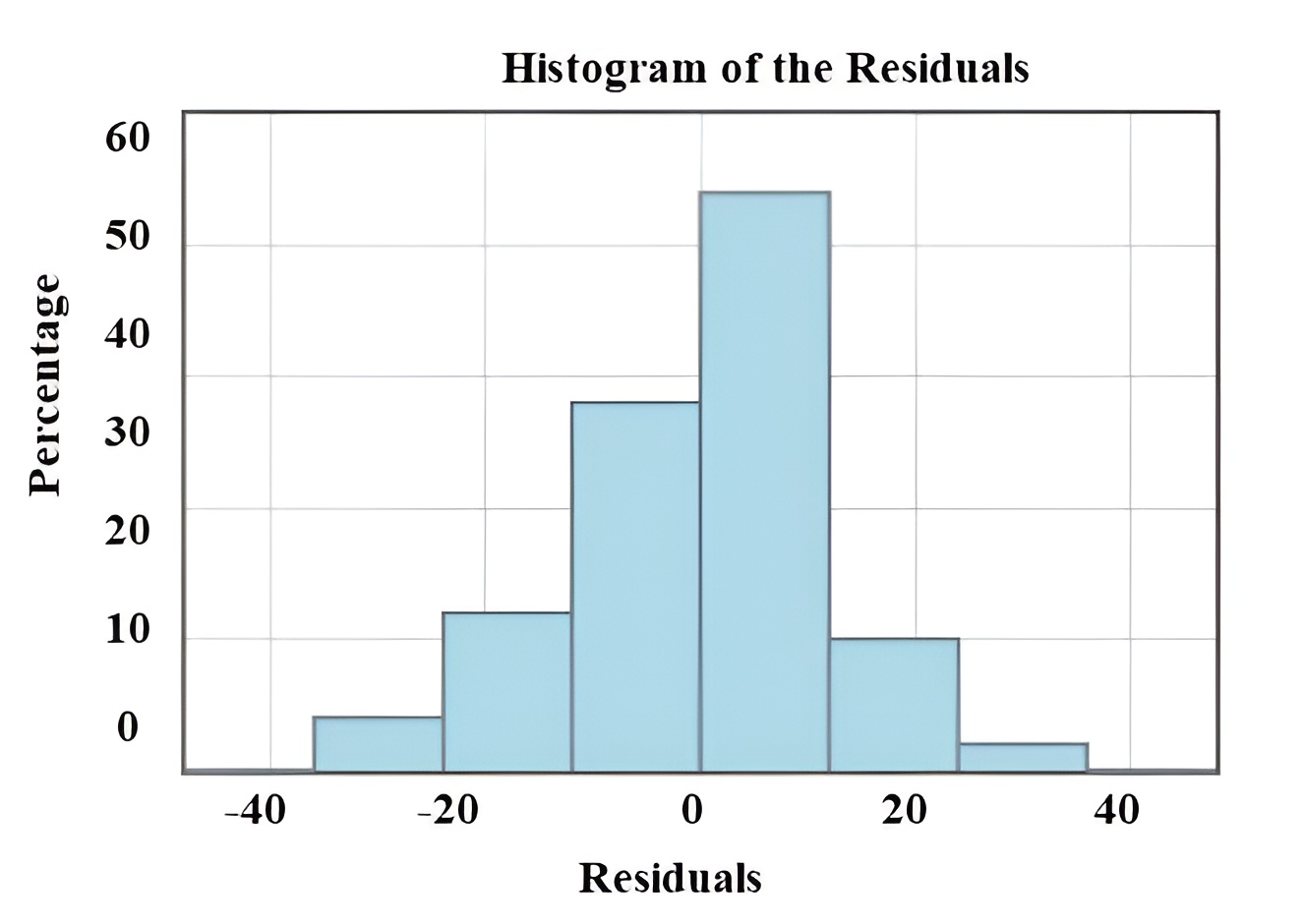

Supplement: Supplemental Information 2 [file peerj-cs-10-2130-s002.zip › Figure S2 Backward Elimination CAM StaffSS/c.jpg]

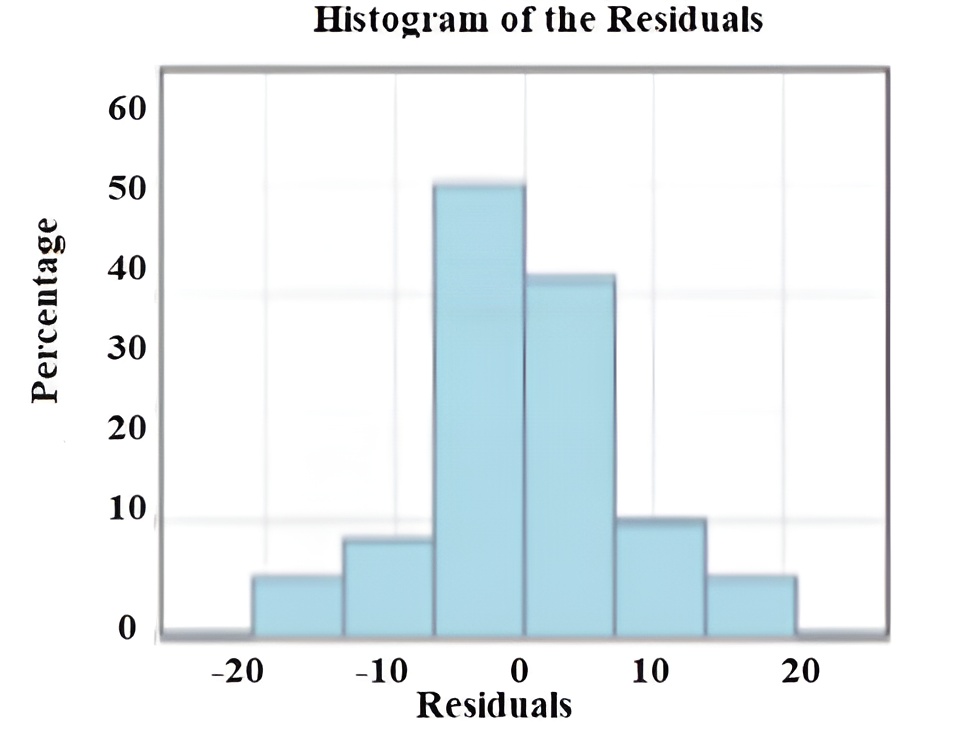

Supplement: Supplemental Information 2 [file peerj-cs-10-2130-s002.zip › Figure S2 Backward Elimination CAM StaffSS/a.jpg]

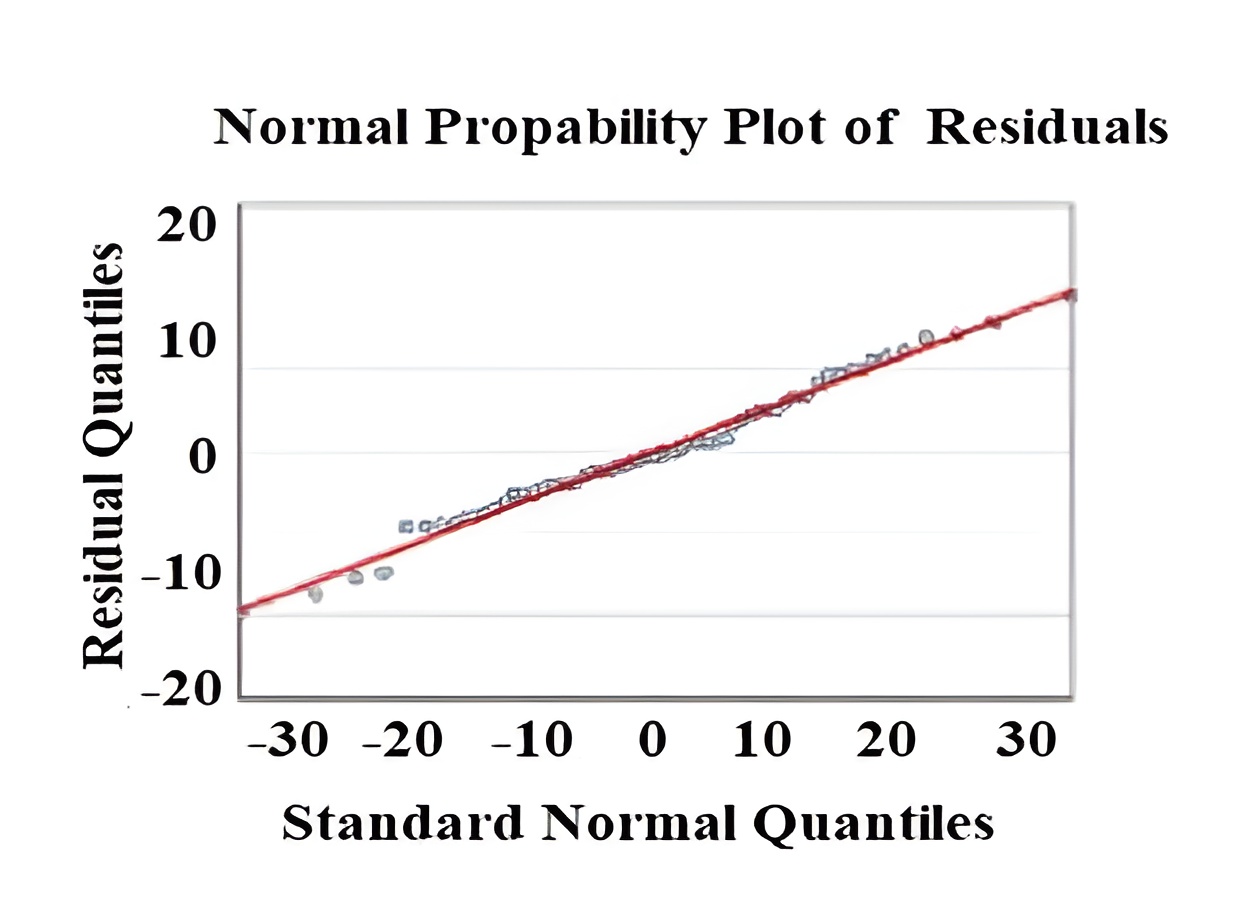

Supplement: Supplemental Information 2 [file peerj-cs-10-2130-s002.zip › Figure S2 Backward Elimination CAM StaffSS/d.jpg]

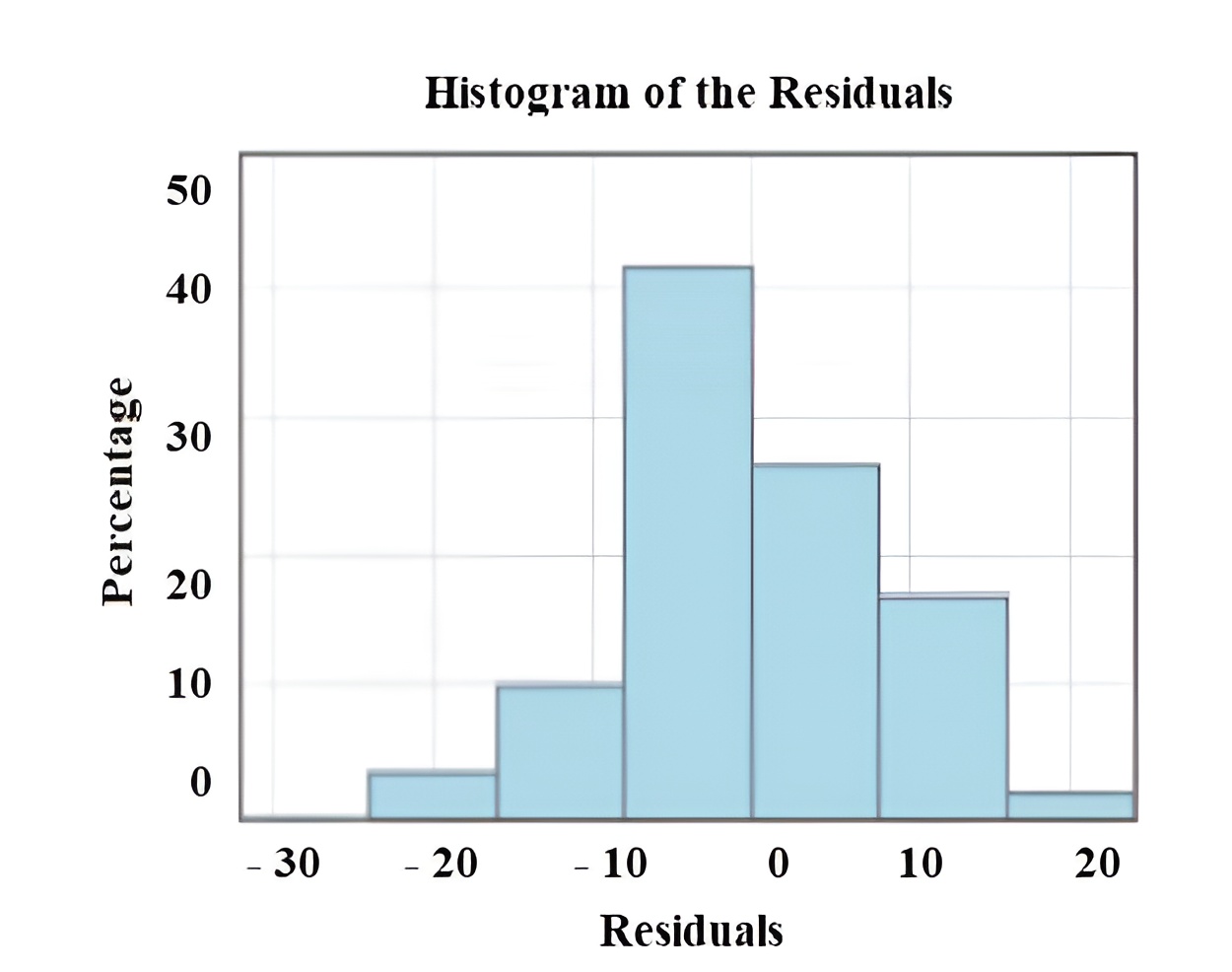

Supplement: Supplemental Information 2 [file peerj-cs-10-2130-s002.zip › Figure S2 Backward Elimination CAM StaffSS/e.jpg]

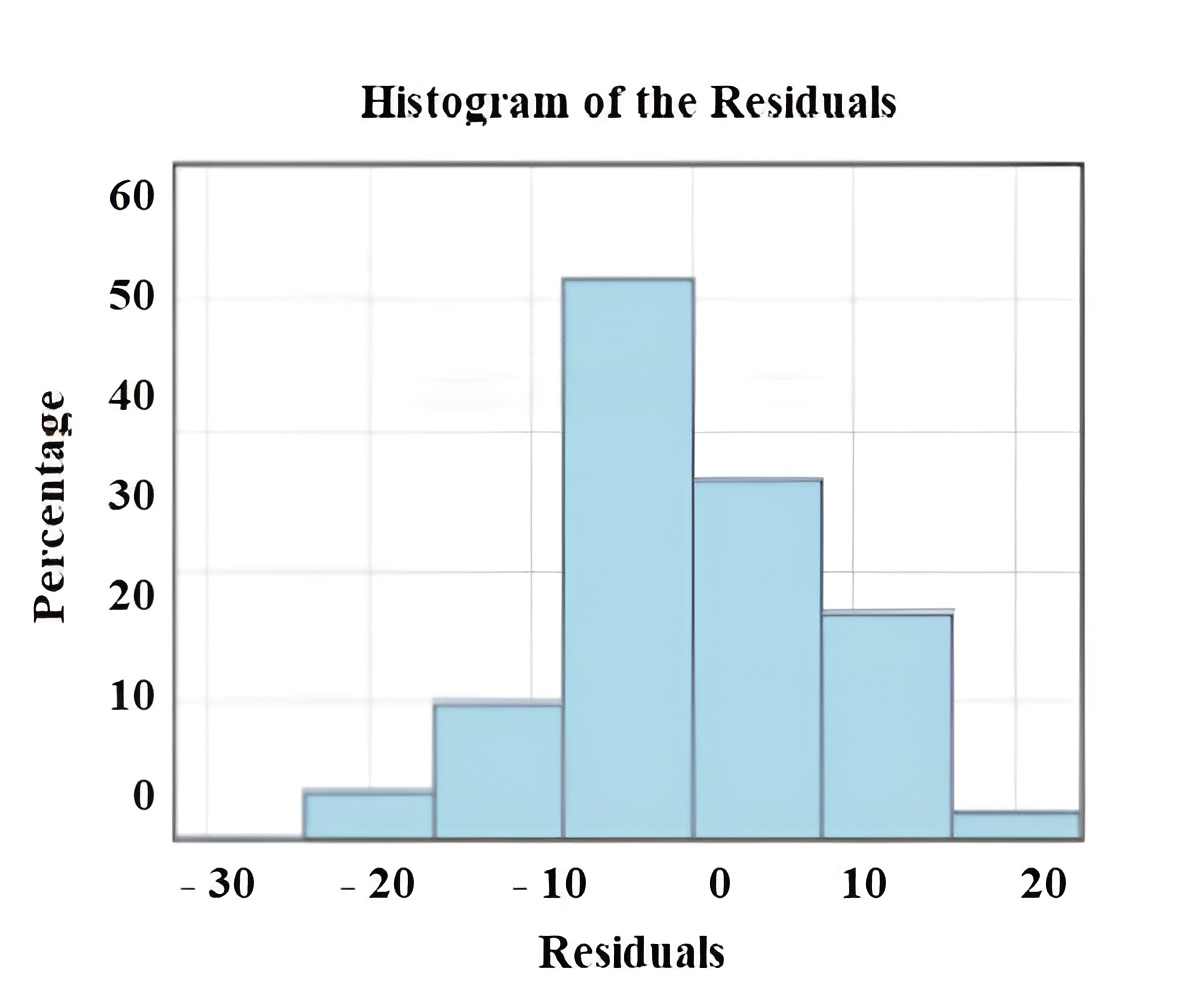

Supplement: Supplemental Information 2 [file peerj-cs-10-2130-s002.zip › Figure S2 Backward Elimination CAM StaffSS/g.jpg]

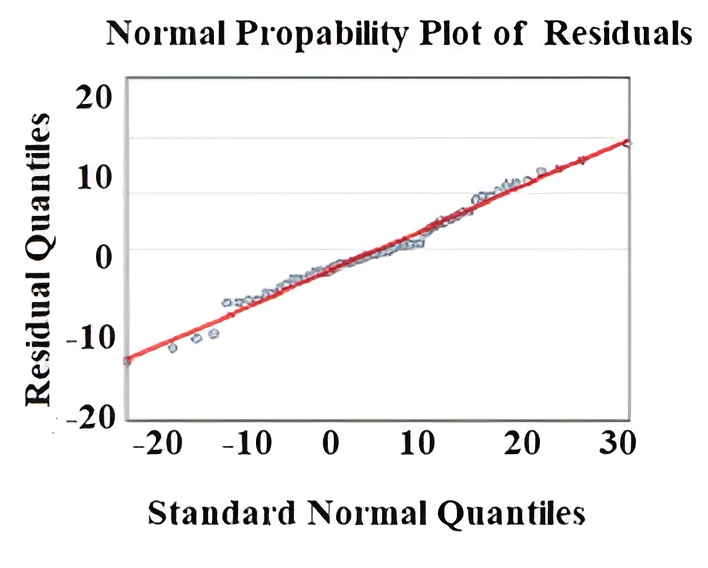

Supplement: Supplemental Information 2 [file peerj-cs-10-2130-s002.zip › Figure S2 Backward Elimination CAM StaffSS/f.jpg]
